# The impact of ^177^Lu-octreotide therapy on ^99m^Tc-MAG3 clearance is not predictive for late nephropathy

**DOI:** 10.18632/oncotarget.9775

**Published:** 2016-06-01

**Authors:** Rudolf A. Werner, Seval Beykan, Takahiro Higuchi, Katharina Lückerath, Alexander Weich, Michael Scheurlen, Christina Bluemel, Ken Herrmann, Andreas K. Buck, Michael Lassmann, Constantin Lapa, Heribert Hänscheid

**Affiliations:** ^1^ Department of Nuclear Medicine, University Hospital Würzburg, Würzburg, Germany; ^2^ Comprehensive Heart Failure Center, University Hospital Würzburg, Würzburg, Germany; ^3^ Department of Internal Medicine II, Gastroenterology, University Hospital Würzburg, Würzburg, Germany; ^4^ Department of Molecular and Medical Pharmacology, David Geffen School of Medicine at UCLA, Los Angeles, CA, United States of America

**Keywords:** renal scintigraphy, MAG3, PRRT, neuroendocrine tumor, ^177^Lu

## Abstract

Peptide Receptor Radionuclide Therapy (PRRT) for the treatment of neuroendocrine tumors may lead to kidney deterioration. This study aimed to evaluate the suitability of ^99m^Tc-mercaptoacetyltriglycine (^99m^Tc­-MAG3) clearance for the early detection of PRRT-induced changes on tubular extraction (TE). TE rate (TER) was measured prior to 128 PRRT cycles (7.6±0.4 GBq ^177^Lu-octreotate/octreotide each) in 32 patients. TER reduction during PRRT was corrected for age-related decrease and analyzed for the potential to predict loss of glomerular filtration (GF). The GF rate (GFR) as measure for renal function was derived from serum creatinine. The mean TER was 234 ± 53 ml/min/1.73 m^2^ before PRRT (baseline) and 221 ± 45 ml/min/1.73 m^2^ after a median follow-up of 370 days. The age-corrected decrease (mean: −3%, range: −27% to +19%) did not reach significance (*p*=0.09) but significantly correlated with the baseline TER (Spearman *p*=−0.62, *p*<0.001). Patients with low baseline TER showed an improved TER after PRRT, high decreases were only observed in individuals with high baseline TER. Pre-therapeutic TER data were inferior to plasma creatinine-derived GFR estimates in predicting late nephropathy. TER assessed by ^99m^Tc-MAG3­clearance prior to and during PRRT is not suitable as early predictor of renal injury and an increased risk for late nephropathy.

## INTRODUCTION

Recent randomized trial results of peptide receptor radionuclide therapy (PRRT) in neuroendocrine tumors (NET) reporting a significant improvement of progression-free survival will ultimately result in increasing clinical use of PPRT [1]. PRRT is highly effective and usually well-tolerated even after repeated treatment cycles [2–4]. However, impairment of renal function even years after initiation of therapy, especially after treatment with ^90^Y conjugates, may become evident [5]. Generally, a safety limit of 23 Gy maximum cumulated absorbed dose to the kidneys is assumed [6].

Since the kidneys represent the dose-limiting organ for PRRT, pre-therapeutic and serial measurements of kidney function using laboratory (e.g., serum creatinine (SCr)) or imaging tests are mandatory [6]. In daily routine, kidney function is usually assessed by estimating the Glomerular Filtration Rate (GFR) from the SCr concentration although such measurements are affected by confounding factors like dietary intake of creatinine [7] or presence of comorbidities like hepatic insufficiency [8] or glomerulopathy [9]. Based on SCr measurements, the decline in renal function after PRRT has been reported to be more pronounced in patients with impaired baseline than in patients with preserved renal function [10]. However, as the decline of GFR must be at least 50% to become evident in patients with initially normal function, SCr based GFR estimates (hereafter referred to as eGFR) are insensitive to early toxicity [11] and underestimate renal impairment in 12% of subjects after PRRT as compared to ^99m^Tc-diethylenetriaminepentaacetic acid (^99m^Tc- DTPA) clearance measurements [12].

It has been suggested that serial assessment of renal function by measuring tubular extraction with ^99m^Tc-mercaptoacetyltriglycine (^99m^Tc-MAG3) or orthoiodohippurate might permit earlier and more sensitive detection of renal damage [12–14]. Renal irradiation from PRRT is most likely to be caused by uptake of radiolabeled somatostatin analogues in the proximal tubules where the radio-peptide is reabsorbed by the megalin receptor and subsequently retained in the interstitium [12, 15]. Hence, the tubular extraction might be a superior indicator for early stages of kidney affection by PRRT [12, 13].

Aiming at an early diagnostic method to identify an increased probability of renal impairment, we retrospectively analyzed ^99m^Tc-­MAG3 clearance data to assess the effect of PRRT on the tubular extraction rate (TER) and to identify parameters of influence and associated risk factors for severe loss of kidney function.

## RESULTS

### Tubular extraction rate (TER) prior to and after PRRT

The mean TER was 234 ± 53 ml/min/1.73 m^2^ at baseline and 221 ± 45 ml/min/1.73 m^2^ at the last assessment after a median of 370 days (quantiles: min, 134 d; 25%, 257 d; 75%, 614 d; max, 915 d). In order to correct for the normal decrease of TER with age, all measured TER values were normalized to the age-adjusted lower limit of the normal range (TER_LoLi_). The median ratio TER/ TER_LoLi_ was 1.36 (quantiles: min, 0.92; 10%, 1.05; 90%, 1.68; max, 2.24) at baseline and 1.27 (quantiles: min, 0.96; 10%, 1.06; 90%,1.56; max, 2.0) after PRRT. Three patients with initially low TER/TER_LoLi_ values (0.92, 0.93, and 0.93; age at primary diagnosis, 63, 70, and 71 y, respectively) showed normalized ratios after PRRT (1.08, 1.05, and 1.11, respectively). All three patients took nephrotoxic medication regularly and two of them underwent chemotherapy prior to PRRT and suffered from diabetes and arterial hypertension.

**Figure 1:**
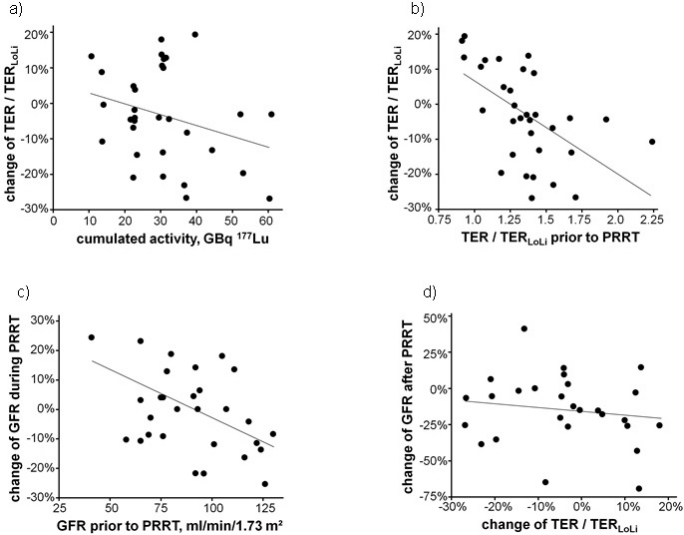
Relative change of the tubular extraction rate (TER) normalized to the lower normal limit TER_**LoLi**_ between the first and last clearance measurements as a function of a the cumulated activity (Spearman ρ = −0.21; *p* = 0.25) and **b.** the ratio TER/TER_LoLi_ prior to Peptide Receptor Radionuclide Therapy (PRRT) (Spearman ρ = −0.62; *p* < 0.001) as well as **c.** relative change of estimated glomerular filtration rate (eGFR) during treatment as a function of the renal function prior to PRRT (Spearman ρ = −0.38; *p* = 0.03) and **d.** the change of eGFR in the follow-up of 27 patients as a function of the change of TER/TER_LoLi_ during PRRT (Spearman ρ = −0.16; *p* = 0.42). The lines indicating linear regressions were drawn to guide the eyes.

Normalized to the baseline ratios, the final mean value of TER/TER_LoLi_ was reduced in the entire cohort to 97% (95% confidence interval of the mean, 92%-102%; standard deviation, 14%). The trend towards reduced renal function did not reach statistical significance (*p* = 0.09; Wilcoxon test). The relative change of the ratio TER/TER_LoLi_ from baseline to the last measurement ranged from −27% to +19% (Figure 1) with 21 patients showing a decreased and 11 an improved TER-ratio. Loss of TER was significantly related to the baseline TER (Spearman ρ = −0.62; *p* < 0.001) but not to the cumulative activities A_cum_, the number of treatment cycles, the treatment duration, the patient age, prior chemotherapy, hypertension, diabetes mellitus, antihypertensive medication, intake of analgesics or somatostatin analogues (Table 1).

**Table 1:** Significance of clinical parameters on the change of TER/TER_LoLi_ during PRRT

Clinical parameters	ρ	*p*	
Baseline TER	−0.62	<0.001	Spearman
Change of glomerular filtration rate during PRRT in follow-up (27 patients)	0.27 -0.16	0.13 0.42	
Cumulative activities, A_cum_	−0.21	0.25	
Number of treatment cycles	−0.09	0.61	
Treatment duration^*^	−0.05	0.81	
Patient age	0.08	0.65	
Prior chemotherapy		0.46	T-test
Hypertension		0.12	
Diabetes mellitus		0.43	
Antihypertensive medication		0.42	
Intake of analgesics		0.46	
Somatostatin analogues		0.36	
Furosemide intake (4 of 32 patients)		0.02	

* Treatment duration = time span between first PRRT cycle and last TER measurement. TER = tubular extraction rate, TER/TER_LoLi_ = TER normalized to the lower normal limit TER_LoLi_, PRRT = Peptide Receptor Radionuclide Therapy.

Spearman's ρ was calculated to test for statistical dependence on a variable, Student's t-test was used to compare groups.

Four patients under furosemide intake showed an increase of TER/TER_LoLi_ to 111% ± 4% of the initial value which was higher than in the rest of the cohort (*p* = 0.02; *T*-test). One patient presented a decrease of TER/TER_LoLi_ under the lower normal limit during therapy from 1.19 to 0.96.

### Regression analyses

**Figure 2:**
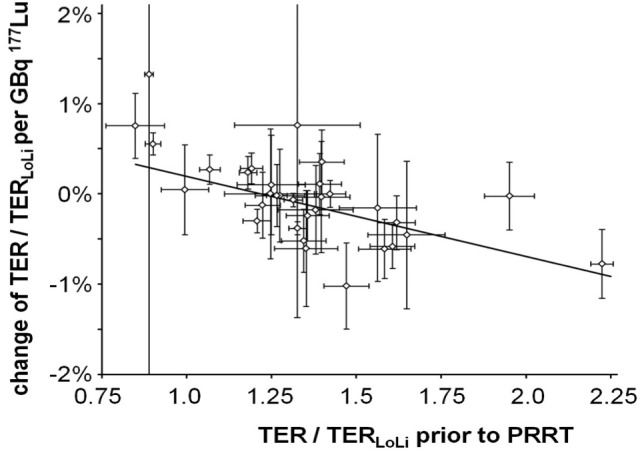
Dependence of the change of tubular extraction rate TER/TER_**LoLi**_ per administered activity in GBq as a function of TER/TER**_LoLi_** prior to first therapy Each data point with error bars represents the result of a regression analysis as illustrated in Figure 3 with the correspondent uncertainty of the regression parameters. Small error bars indicate a clear trend of TER/TER_LoLi_ with minor scatter of data. The result of a weighted linear regression to the data is shown by a straight line characterized by intercept 1.08% ± 0.27% and slope −0.89% ± 0.20%. Individuals with typical renal clearance TER-1.43 TER_LoLi_ are expected to have a relative loss of TER of 0.20% per GBq [^177^Lu-DOTA(0),Tyr(3)]-octreotate/octreotide (^177^Lu-DOTATATE/-TOC) or 1.4% per treatment cycle with a standard activity of 7.5 GBq ^177^Lu-DOTATATE/-TOC. TER = tubular extraction rate, TER/TER_LoLi_ = TER normalized to the lower normal limit TER_LoLi_, PRRT = Peptide Receptor Radionuclide Therapy.

In order to demonstrate the dependence of the change of TER/TER_LoLi_ per administered activity in GBq as a function of TER/TER_LoLi_ prior to first therapy, regression analyses of TER/TER_LoLi_ *vs*. administered activities was performed (Figure 2). The change of TER/TER_LoLi_ per GBq ^177^Lu-DOTATATE/-TOC was significantly correlated to the initial renal function (Spearman ρ = −0.62; *p* < 0.001).

### TER and kidney uptake

Twenty-five of the 32 patients had single photon emission computed tomography/computed tomography (SPECT/CT)data acquired at 24 h after the activity administration in at least 2 treatment cycles. The uptake per ml of kidney parenchyma increased with decreasing ratio TER/TER_LoLi_ (ρ = −0.24; *p* = 0.01; Spearman; Supplementary Figure 1a). Loss of TER in the course of treatments was associated with increasing kidney uptake as assessed by intra-individual mean change of uptake per ml of kidney parenchyma from treatment cycle to treatment cycle as a function of the change of TER (ρ = −0.43; *p* < 0.03; Spearman; Supplementary Figure 1b).

### Estimated GFR (eGFR) prior to and after PRRT

The mean eGFR was 92 ± 25 ml/min/1.73 m^2^ prior to PRRT and 90 ± 22 ml/min/1.73 m^2^ at the end of the last treatment cycle; the reduction was not statistically significant (ρ = 0.48; paired samples *T*-test). At the end of therapy, eGFR was higher in 15, lower in 14, and unchanged in 3 individuals. Change of eGFR during treatment was significantly correlated to the initial renal function (Spearman ρ = −0.38; *p* = 0.03; Figure 1c). More than 10% loss of eGFR was only observed in patients with good renal function (> 90 ml/min/1.73 m^2^). The correlation observed between the change of eGFR and the change of TER/TER_LoLi_ during PRRT was not significant (Table 1).

As compared to the measurements at the end of treatment, the eGFR was significantly reduced to 78 ± 34 ml/min/1.73 m^2^ (*p* = 0.01; paired samples *T*-test) in follow-up measurements of the serum creatinine performed in 27 of the 32 patients at a median of 837 days after the PRRT initiation (quantiles: min, 158 d; 25%, 632 d; 75%, 955 d; max, 1825 d). Follow-up eGFR data were lower in 20, unchanged in 1, and higher in 6 individuals. According to the Common Terminology Criteria for Adverse Events (CTCAE) 4.0, grade 2 toxicity was observed in 5 of the 27 patients (18.5%). One individual with reduced eGFR prior to (58 ml/min/1.73 m^2^, TER/TER_LoLi_: 0.93) and after (52 ml/min/1.73 m^2^, TER/TER_LoLi_: 1.05) three treatment cycles (total 17.9 GBq ^177^Lu-DOTATATE/-TOC) showed grade 3 toxicity (1/27, 3.7%) at follow-up 1818 days after start of PRRT. This patient did not receive any additional therapy after PRRT nor experienced disease progression within follow-up. However, a potential nephrotoxic chemotherapy regimen including cisplatin and fluorouracil prior to PRRT was performed. The change of TER during PRRT was not predictive for late kidney failure (Figure 1d).

The relative loss of eGFR during follow-up after PRRT correlated better with the pre-therapeutic eGFR (Spearman ρ = 0.61; *p* = 0.001) than with the pre-therapeutic TER/TER_LoLi_ ratio (Spearman ρ = 0.38; *p* = 0.05). No correlation was observed with the change of TER/TER_LoLi_ (Spearman ρ = −0.16; *p* = 0.42, Figure 1d) and the change of eGFR (Spearman ρ = −0.28; *p* = 0.16) during PRRT.

Twenty of the patients with eGFR follow-up had SPECT/CT data acquired at 24 h after the activity administration in at least 2 treatment cycles. The relative loss of eGFR during follow-up was not associated with the mean kidney uptake in these patients (ρ = −0.06; *p* < 0.79; Spearman; Supplementary Figure 1c).

In this cohort, pre-therapeutic TER data were inferior to GFR estimates based on plasma creatinine in predicting radiation-induced nephropathy after PRRT. The change of TER during PRRT was not predictive for late toxicity.

## DISCUSSION

This is the first study to assess PRRT-induced renal toxicity by serial ^99m^Tc-MAG3 clearance measurements. A mean TER loss of 3% between the first and the last assessment was recorded. This decline in kidney function is lower than previously reported for creatinine clearance with *Gupta et al.* reporting a significant decrease of 24% in GFR and a major increase in SCr after ^177^Lu-octreotate [10]. *Valkema et al.* demonstrated GFR reductions of 4-7% per year for ^177^Lu-octreotate and ^90^Y-octreotide [16]. Also investigating GFR but measuring GFR more accurately by use of ^99m^Tc-DTPA, *Sabet et al*. observed minor renal impairment in 43% of the cohort and serious renal adverse effects occurring in only 1.3% after ^177^Lu-octreotate therapies [12]. The yearly loss of GFR reported by *Sabet et al.* of 2% is similar to our findings of an age-corrected loss of TER of 3%. As in the present study, clinical parameters like patient age, prior chemotherapy, diabetes mellitus, antihypertensive medication or cumulated administered activity have reported not to be associated with loss of function [12].

However, the most remarkable observation was the significant dependence of TER reduction on the renal function prior to therapy: only patients with initially high clearance showed a considerable loss of TER. In contrast, patients with low TER experienced no further decrease. In line with our findings, *Garske et al.* also observed the same phenomenon of improvement of renal function under PRRT and therefore recommended to estimate kidney function from more than one test to avoid false positive results. The reason for this observed improvement is not totally clear: It was hypothesized that the GFR improvement could also be seen in the light of better urinary flow due to reduced tumor burden in the case of shrinkage [17]. However, none of the investigated patients in our study experienced a significant reduction of tumor mass.

Neither the cumulative activities were decisive for TER reduction in our cohort (data not shown) nor was higher activity uptake in the kidneys associated with higher loss of TER. On the contrary, a significant negative correlation was observable between MAG3 clearance and renal uptake at 24 h after activity administration. A similar correlation has recently been found by *Svensson et al*. who reported that renal absorbed doses appeared to be higher in patients with baseline impaired renal function [18]. Interestingly, loss of eGFR in the follow-up was not associated with kidney uptake during therapy.

Although dose estimates deduced from the SPECT/CT uptake data of our patients and the mean half-life reported by *Garske et al.* [19] suggest that all but one patient remained below the safety limit of 23 Gy kidney absorbed dose, late GFR follow-up measurements revealed grade 2/3 toxicity in 6 of 27 (22.2%) patients. This finding is in line with a study including 290 patients after ^177^Lu-based PRRT which detected grade 1/2 renal toxicity in 25.5% [20]. It should, however, be noted that our data cannot be used to demonstrate the toxicity of PRRT with ^177^Lu because most of the patients in our cohort received other potentially nephrotoxic treatment as well. Late clinical manifestation of renal toxicity was not related to loss of TER during PRRT but correlated with renal function prior to therapy. The eGFR prior to therapy, although deduced from SCr measurements only, correlated better with the relative loss of eGFR after PRRT than the TER prior to treatment. The ^99m^Tc-MAG3 clearance assessments performed in our patients prior to and during PRRT demonstrated only minor loss of renal function during PRRT, an observation which was supported by the minor changes of eGFR observed during treatment. Relevant information was not gained from the TER measurements which were unsuitable as early diagnostics to identify an increased probability of later renal failure. Moreover, blood-based GFR estimation of kidney function has the advantage of being more simple, rapid and less expensive as compared to ^99m^Tc-MAG3 studies.

This study has some limitations. After the last TER assessment, 13 patients (40%) received another treatment cycle which might have also influenced renal function during long-term follow-up. The statistical power is limited by the small patient number, the retrospective nature of the analysis, and the lack of information on the effective half-lives of the activity in the kidneys preventing reliable assessments of the renal absorbed doses. A prospective study including full kidney dosimetry and a larger cohort with more patients with renal impairment is desirable to strengthen our preliminary findings. Furthermore, other assessments of tubular function, such as alpha-1 microglobulin (urine), and direct comparison to exactly measured glomerular function e.g. by ^99m^Tc-DTPA might provide further insight into the pathological process of PRRT-related nephrotoxicity.

## MATERIALS AND METHODS

### Study design

Since this study comprised the retrospective analysis of routinely acquired data, the local ethics committee waived the need for further approval. All patients gave written informed consent for the recording and anonymized analysis of their data.

### Patient cohort

Thirty-two patients (7 female, 25 male) with histologically proven advanced NET who underwent PRRT at our institution between April 2010 and July 2014 were retrospectively analyzed. Patients received at least 3 PRRT treatment cycles up to July 2015. Except 2 young patients (aged 24 and 30 years, respectively), the mean age of included patients at start of therapy was 64.7 ± 9.0 years. All patients gave written informed consent to the therapeutic and diagnostic procedures. Detailed patient information is provided in Table 2. Prior to the first PRRT cycle, somatostatin receptor-directed positron emission tomography (PET) was performed to prove receptor expression by the tumor in each individual. The general exclusion criteria, as defined by the *Joint IAEA, EANM, and SNMMI practical guidance*, were applied [6]. Kidney function measurements prior to each PRRT cycle were performed to verify the TER to be at least 60% of the mean age-adjusted normal value. Pre-existing risk factors for the occurrence of kidney toxicity such as diabetes mellitus (*n* = 6), arterial hypertension (*n* = 13), nephrotoxic drug intake, long-term use of analgetics, antihypertensive, furosemide and lipid-lowering medication (*n* = 16) were recorded.

**Table 2:** Patients' characteristics

Characteristic		Number of patients (%)
Sex	female male	7/32 (21.9) 25/32 (78.1)
Primary	small intestine pancreatic unknown lung thyroid other	10/32 (31.3) 9/32 (28.1) 2/32 (6.25) 2/32 (6.25) 2/32 (6.25) 7/32 (21.9)
Previous treatment	somatostatin analogues chemotherapy surgery of the primary, transarterial chemoembolization	13/32 (40.6) 8/32 (25) 16/32 (50)
Clinical risk factors	arterial hypertension diabetes mellitus nephrotoxic drugs ^*^	13/32 (40.6) 6/32 (18.8) 16/32 (50)

* analgetics, antihypertensive medication, lipid-lowering medication, furosemide intake.

### Assessment of kidney function

Prior to each treatment cycle, all patients underwent (as part of clinical routine work-up) renal scintigraphy with ^99m^Tc-labelled MAG3 on a single head gamma camera (Signature; Siemens, Erlangen, Germany) equipped with a medium-energy, high-resolution collimator as previously described [21]. TER was assessed from measured activity concentrations in plasma samples corrected for the body surface according to the single sample method introduced by *Bubeck et al.* [22]. Clearance values deduced from samples taken after 20 and 30 min after the ^99m^Tc-MAG3 administration were averaged to minimize errors. Age-adjusted mean normal values TER_Norm_ were calculated according to the formula: TER_Norm_ = (435 − 3.03 x age (y)) ml/min/1.73m^2^ with the lower limit of the normal range: TER_LoLi_ = 70% TER_Norm_ [23].

During and after treatment, serum creatinine (SCr) was measured periodically and the glomerular filtration rate glomerular filtration rate (eGFR) was calculated by the CKD-EPI equation taking age, ethnicity, and weight into account [24].

### Treatment

All patients treated by PRRT complied with the requirement TER> 60% TER_Norm_ as recommended in the guideline [6]. The patients were hospitalized for 3 days starting 1 day prior to therapy to guarantee adequate hydration (1 l saline/day). The radiopharmaceutical, [^177^Lu-DOTA(0),Tyr(3)]-octreotate/octreotide (^177^Lu-DOTATATE/-TOC; ^177^Lu delivered by ITG, Munich, Germany), was prepared as previously reported [25] and infused over 20-30 minutes with a specific activity of 100-150 GBq/μmol. For kidney protection, a solution containing 25 g of lysine and 25 g of arginine diluted in 2 l of normal saline was infused over 4 h, starting 1 h before PRRT [6]. In total, 32 patients received 128 treatment cycles (2, 3, 4, 5, 6, 7, and 8 cycles in 5, 9, 10, 3, 1, 2, and 2 patients, respectively) with subsequent TER evaluation. After the last TER assessment, 13 of the patients received another treatment cycle without following MAG3 clearance assessment. 124 treatment cycles included in the analysis were performed with standard activities of 7.6±0.4 GBq ^177^Lu-DOTATATE/-TOC. One patient with slightly decreased renal function of TER- 65% TER_Norm_ was treated twice with reduced activities of 5.2 and 5.5 GBq ^177^Lu-DOTATATE. The last cycle of this patient (7.2 GBq ^177^Lu-DOTATATE) without subsequent TER assessment was excluded from TER analysis. Another patient with TER = 109% TER_Norm_ received two compassionate use treatment cycles with activities of 19.3 and 17.3 GBq ^177^Lu-DOTATOC and an unconsidered last cycle with 8.1 GBq ^177^Lu-DOTATOC (without subsequent TER assessment). The median time interval between treatment cycles was 91 days (range: 63 - 830 d; 1^st^ quartile: 84 d, 3^rd^ quartile: 112 d).

An exact assessment of kidney absorbed doses from therapy was not possible because serial images necessary to determine the effective half-life in the organ had not been acquired. However, SPECT/CT of the abdomen was performed 24 h (*n* = 101) or 2 to 5 days (*n* = 18) after activity administration in 119/128 treatment cycles. All SPECT/CT images were acquired using the same double head gamma camera (Siemens Symbia T2; Siemens, Erlangen, Germany) equipped with medium energy collimators calibrated by phantom measurements with ^177^Lu activity standards (sensitivity, sum of both heads: 28 counts per second per MBq ^177^Lu in a 15% window at 208 keV). Data were reconstructed using a 3D Ordered Subsets Expectation-Maximization (OSEM, 6 subsets, 6 iterations, Gauss 6 mm) algorithm with corrections for scatter and attenuation to obtain absolute activity quantification in voxels sized 0.11 cm^3^.

### Data evaluation

To identify parameters influencing TER, all TER data deduced from ^99m^Tc-MAG3 clearance measurements were normalized to the lower limit of normal range by calculating the ratio TER/TER_LoLi_. This method corrects for the normal decrease of renal function with age in the course of treatment for the individual patient and makes data from individuals of different ages comparable. The ratio TER/TER_LoLi_ is 1 or larger in patients with normal kidney function, < 1 in those with impaired TER, and is expected to equal 1/70% = 1.43 in individuals with a typical renal clearance TER_Norm_ matching the mean age-adjusted normal value.

The time course of TER/TER_LoLi_ was analyzed for each patient by comparing the MAG3 clearance data measured at baseline and after the last PRRT cycle and relating the change of TER/TER_LoLi_ to the activities administered between both assessments of renal function. As explained above, data from the last PRRT cycle without later TER measurement were omitted from analysis.

**Figure 3:**
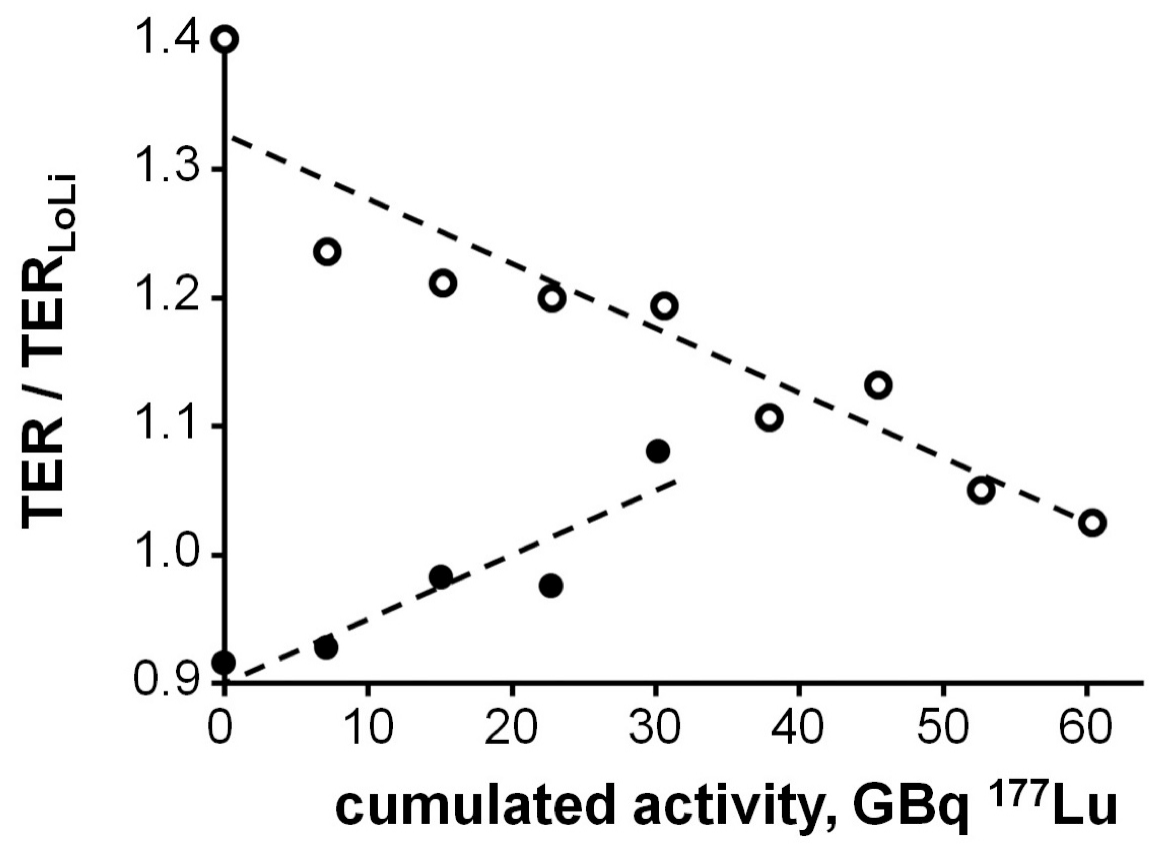
Tubular extraction rate (TER) normalized to the lower normal limit (TER_**LoLi**_) *vs* the cumulated activity (A_cum_) in a patient with improving renal function in the course of 4 cycles of Peptide Receptor Radionuclide Therapy (dots) and a patient with loss of function during 8 cycles (circles). The dashed lines show the results of linear regression: TER/TER_LoLi_ = ß_0_+ß_1_*A_cum_ [GBq] (ß_0_: 90.1% ± 2.3% and 132.7% ± 2.7%; ß_1_: 0.50% ± 0.12% and −0.51% ± 0.07% per GBq, respectively). TER/TER_LoLi_ is 1 or larger in patients with normal kidney function and < 1 in those with impaired TER.

In a more complex evaluation, regression analyses including all TER measurements as a function of the previous administered activities were performed for each individual. Examples of linear regressions of TER/TER_LoLi_ *vs*. the cumulated activities A_cum_ (TER/TER_LoLi_ = ß_0_+ß_1_*A_cum_ [GBq]) are shown in Figure 3. The coefficient ß_0_ represents a fit estimate of TER/TER_LoLi_ prior to the first PRRT, ß_1_ the absolute change, and ß_1_/ß_0_ the relative change of TER/TER_LoLi_ per GBq administered activity. The uncertainties of the coefficients, ~ß_0_ and ~ß_1_, respectively, indicate the scatter of the measured TER values.

To determine the dependence of the relative change of TER/TER_LoLi_ on the initial value of TER/TER_LoLi_ prior to PRRT, the values of ß_1_/ß_0_ were calculated for each individual as a function of the corresponding ß_0_ assuming uncertainties ~ß_1_/ß_0_ and ~ß_0_/ß_0_, respectively, and a weighted linear regression was performed.

### Statistical analysis

Statistical analysis was performed using IBM SPSS (version 23.0, Ehningen, Germany). Data were analyzed with non-parametric tests when the Shapiro-Wilk test indicated incompatibility with normal distribution. Spearman's rank correlation coefficient was calculated to test for statistical dependence between two variables. Quantitative values were reported as mean ± standard deviation or median and quantiles as appropriate. The tests used are reported together with the results. All statistical tests were performed two-sided and a *p*-value < 0.05 was considered to indicate statistical significance.

## SUPPLEMENTARY MATERIALS FIGURES


