# Supplementary material for: The impact of 177Lu-octreotide therapy on 99mTc-MAG3 clearance is not predictive for late nephropathy
Source: Oncotarget. 2016 Jun 1;7(27):41233–41. doi: 10.18632/oncotarget.9775 (PMC5173054; doi:10.18632/oncotarget.9775)

The impact of ^{177}Lu-octreotide therapy on ^{99m}Tc-MAG3 clearance is not predictive for late nephropathy

Supplementary Material

Supplementary Figure.

Twenty-five of the 32 patients had SPECT/CT data acquired at 24 h after the activity administration in at least 2 treatment cycles. The uptake per ml of kidney parenchyma increased with decreasing ratio TER/TER_{LoLi} (ρ=-0.24; p=0.01; Spearman; Supplementary Figure a). Loss of TER in the course of treatments was associated with increasing kidney uptake as assessed by intra-individual mean change of uptake per ml of kidney parenchyma from treatment cycle to treatment cycle as a function of the change of TER (ρ=-0.43; p<0.03; Spearman; Supplementary Figure b).

a) b)

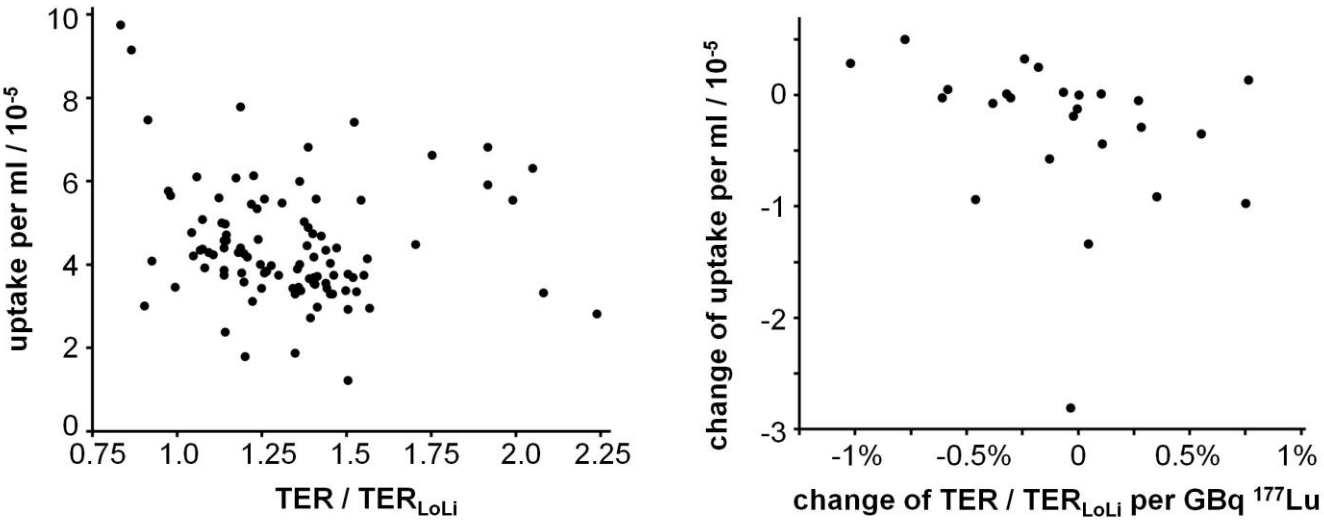

a) Dependence of the fractional activity uptake per ml of kidney parenchyma on the ratio TER/TER_{LoLi} prior to the administration in 101 treatment cycles with SPECT/CT at 24 h.

b) Mean change of uptake per ml of kidney parenchyma between treatment cycles as a function of the change of TER/TER_{LoLi} per administered activity in 25 patients.

SPECT/CT = single photon emission computed tomography/ computed tomography. TER = tubular extraction rate, TER/TER_{LoLi} = TER normalized to the lower normal limit TER_{LoLi}.

Twenty of the patients with GFR follow-up had SPECT/CT data acquired at 24 h after the activity administration in at least 2 treatment cycles. The relative loss of GFR during follow-up was not associated with the mean kidney uptake in these patients (ρ=-0.06; $p<0.79$; Spearman; Supplementary Figure c).

c)

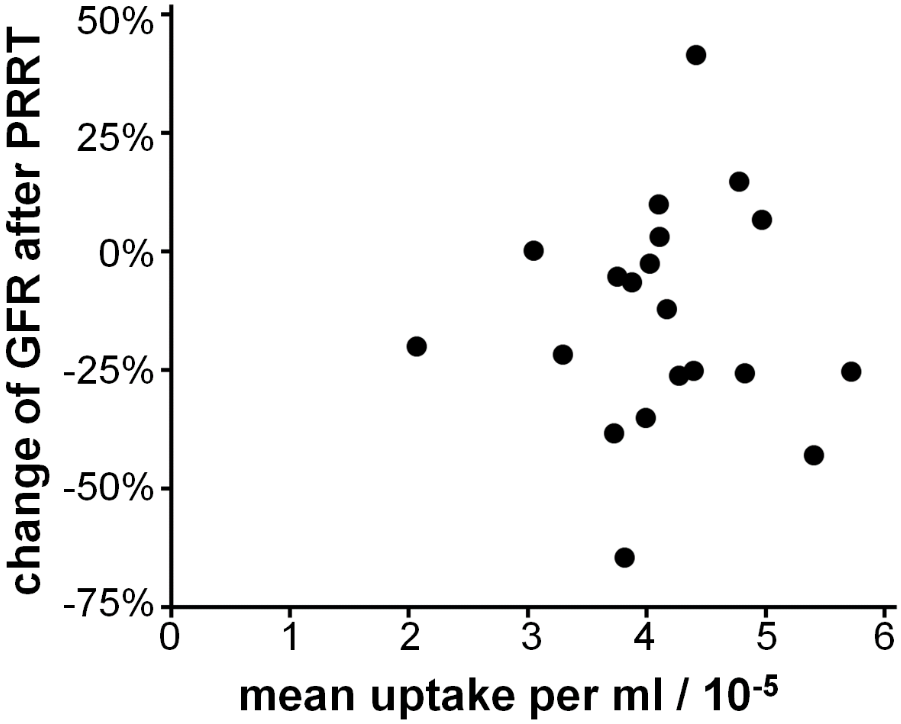

Supplement: Supplementary file 1 [file oncotarget-07-41233-s001.pdf]
